# Chemical Oxidative Polymerization of Methylene Blue: Reaction Mechanism and Aspects of Chain Structure

**DOI:** 10.3390/polym13132188

**Published:** 2021-06-30

**Authors:** Yaroslav O. Mezhuev, Igor Y. Vorobev, Ivan V. Plyushchii, Efrem G. Krivoborodov, Alexander A. Artyukhov, Mikhail V. Motyakin, Anna L. Luss, Irina S. Ionova, Alexander L. Kovarskii, Igor A. Derevnin, Valerie A. Dyatlov, Ruslan A. Alekperov, Ilya Y. Toropygin, Mikhail A. Volkov, Mikhail I. Shtilman, Yuri V. Korshak

**Affiliations:** 1 Department of Biomaterials, Mendeleev University of Chemical Technology of Russia, 125047 Moscow, Russia; eterenos@gmail.com (I.Y.V.); 12345bob@mail.ru (I.V.P.); vv1992@yandex.ru (E.G.K.); artyukhow@yandex.ru (A.A.A.); al.luss@yandex.ru (A.L.L.); derevninigor616@yandex.ru (I.A.D.); dyatlov.va@mail.ru (V.A.D.); alekperov2016@gmail.com (R.A.A.); shtilmanm@yandex.ru (M.I.S.); yukorshak@yandex.ru (Y.V.K.); 2 Emanuel Institute of Biochemical Physics, Russian Academy of Sciences, 119334 Moscow, Russia; motyakin@hotmail.com (M.V.M.); alkogon383@yandex.ru (A.L.K.); 3 Semenov Federal Research Center for Chemical Physics, Russian Academy of Sciences, 119991 Moscow, Russia; ionova63is@yandex.ru; 4 V.N. Orekhovich Research Institute of Biomedical Chemistry, Russian Academy of Medical Sciences, 119832 Moscow, Russia; toropygin@rambler.ru; 5 A.N. Frumkin Institute of Physical Chemistry and Electrochemistry of the Russian Academy of Sciences, 119071 Moscow, Russia; mendeleev93@yandex.ru

**Keywords:** oxidative polymerization, methylene blue, polymethylene blue, electrical conductivity of polymers, paramagnetic polymers

## Abstract

The kinetic regularities of the initial stage of chemical oxidative polymerization of methylene blue under the action of ammonium peroxodisulfate in an aqueous medium have been established by the method of potentiometry. It was shown that the methylene blue polymerization mechanism includes the stages of chain initiation and growth. It was found that the rate of the initial stage of the reaction obeys the kinetic equation of the first order with the activation energy 49 kJ · mol^−1^. Based on the proposed mechanism of oxidative polymerization of methylene blue and the data of MALDI, EPR, and IR spectroscopy methods, the structure of the polymethylene blue chain is proposed. It has been shown that polymethylene blue has a metallic luster, and its electrical conductivity is probably the result of conjugation over extended chain sections and the formation of charge transfer complexes. It was found that polymethylene blue is resistant to heating up to a temperature of 440 K and then enters into exothermic transformations without significant weight loss. When the temperature rises above 480 K, polymethylene blue is subject to endothermic degradation and retains 75% of its mass up to 1000 K.

## 1. Introduction

The ability to control the properties and morphology of thin films of electroactive polymethylene blue [1] during the electrochemical polymerization of methylene blue [2] opens up prospects for the use of this polymer. Although research aimed at establishing the laws of synthesis and properties of polymethylene blue is still not enough, it has already found application for the manufacture of electrochemical sensors. For example, using polymethylene blue, it has been shown that it is possible to determine the nitrite ion [3], 4-nitrophenol [4], acrylamide [5], and thiols and amino acid derivatives [6]. The successful use of polymethylene blue for the detection of natural substances, such as catechins in green tea [7], pyridoxine [8], DNA [9], hemoglobin [10], and xanthan and uric acid [11], give hope for the creation of new analyzers for medical and biological purposes. The methylene blue polymer can act as a trap for hydroxyl radicals [12], is an effective adsorbent [13], and is promising for creating supercapacitors [14]. Films based on polymethylene blue have a high conductivity of about 103 S · cm^−1^, which is of interest for the creation of conducting glasses [15]. Polymethylene blue can also be synthesized by chemical oxidation of methylene blue by the action of ammonium peroxodisulfate [9].

Although the area of possible application of polymethylene blue is rapidly expanding, the chain structure of this polymer remains a matter of debate [5,7,15,16,17]. The mechanism of photopolymerization of unsaturated compounds in the presence of methylene blue and reducing agents was described earlier [18,19], but the details of the mechanism of chemical oxidative polymerization of the methylene blue dye itself have not been established [17]. Therefore, this article aims to establish the kinetics and mechanism of chemical oxidative polymerization of methylene blue, as well as to consider the chain structure, thermal stability, and the nature of paramagnetism of polymethylene blue. In this work, based on the analysis of the results of MALDI, EPR, IR spectroscopy, and kinetic data, it is proposed that the mechanism of oxidative polymerization includes the stage of conversion of molecular dimers of methylene blue (stabilized by charge transfer) into radical cations of covalent dimers of methylene blue, which initiate the subsequent addition of the monomer.

## 2. Experimental

### 2.1. Materials and Methods

Methylene blue and ammonium peroxodisulfate were purchased from Sigma–Aldrich. Double-distilled water was used as a reaction medium. Vibrational spectra of the samples were recorded in potassium bromide pellets using a Nicolet 380 spectrometer (Thermo Fisher Scientific, Waltham, MA, USA). MALDI spectra were recorded on an Ultraflex II mass spectrometer (Bruker, Karlsruhe, Germany) with an accelerating voltage of 25 kV with Nd: YAG laser (355 nm) desorption from a DHB matrix. EPR spectra of solid samples of methylene blue and polymethylene blue were recorded on a commercial spectrometer Bruker EMX (Bruker, Karlsruhe, Germany) (For EPR measurements, we used the devices of the Core Facility of the Emanuel Institute of Biochemical Physics, Russian Academy of Sciences “New Materials and Technologies”). Photographs of methylene blue samples and the product of its oxidative polymerization were obtained using a stereoscopic microscope Lomo MSP-2-2SD equipped with an MC-5 electronic camera (Lomo Microsystems, St. Petersburg, Russia); images were processed using the MCview (Lomo Microsystems, St. Petersburg, Russia) software package. TGA and DSC were recorded at a heating rate of 10 degrees · min^−1^ in a nitrogen flow (70 mL · min^−1^) on an STA 449 F3 Yupiter instrument (NETZSCH, Weyhe-Dreye, Germany). XRD was recorded using a Malvern Panalytical Aeris XRD benchtop X-ray diffractometer (Malvern, Worcestershire, UK).

### 2.2. Synthesis of Polymethylene Blue

An amount of 0.320 g of methylene blue was dissolved in 50 mL of bidistillate (a CODYSON CDS-300 17269 ultrasonic bath (Codyson, Moscow, Russia) was used to accelerate the dissolution). A solution of 0.912 g of ammonium peroxodisulfate in 50 mL of bidistillate was also prepared. The solutions were mixed, and the reaction was carried out at a temperature of 298 K for 18 h. The resulting precipitate was filtered off, washed on a filter with 25 portions of water, 100 mL each, and then dried at a temperature of 323 K.

### 2.3. Kinetics of Polymerization of Methylene Blue

An amount of 0.160 g of methylene blue was dissolved in 50 mL of bidistillate. A solution of 0.456 g of ammonium peroxodisulfate was also prepared and dissolved in 50 mL of bidistillate. The electrode of an F20-Standard pH meter manufactured by Mettler Toledo was placed in an aqueous solution of a monomer, and a solution of an oxidizing agent was quickly added, recording the pH values at specified time intervals. Kinetic studies were carried out under thermostating conditions at temperatures of 298 K, 308 K, and 318 K.

## 3. Results and Discussion

**Figure 1:**
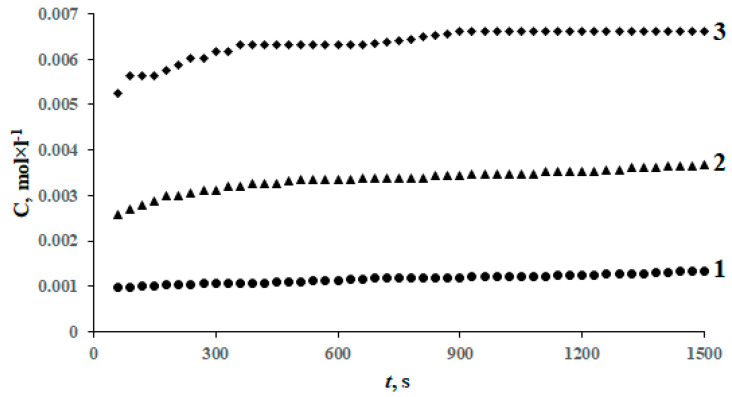
Kinetic curves of methylene blue polymerization at the following temperatures: 1–298 K; 2–308 K; 3–318 K (methylene blue concentration 5 · 10^−3^ mol · L^−1^; concentration of ammonium peroxodisulfate 0.02 mol · L^−1^).

The time dependences of proton concentrations obtained under the conditions of oxidative polymerization of methylene blue at different temperatures are shown in Figure 1.

After a short period of rapid increase in the concentration of protons, the rate of their release decreases significantly (Figure 1). In this case, it takes at least several hours to complete the reaction with the formation of an insoluble polymer. Thus, a significant part of methylene blue is rapidly oxidized, followed by a slower stage associated with the formation of a water-insoluble polymer. The time boundaries of the stage of rapid oxidation of the monomer, presumably with the formation of a dimer, are clearly traced (Figure 1) and are about 750, 510, and 360 min at temperatures of 298 K, 308 K, and 318 K, respectively. By the indicated times, the concentration of protons in the system is 1.19 · 10^−3^, 3.52 · 10^−3^, and 6.31 · 10^−3^ mol · L^−1^ at temperatures of 298 K, 308 K, and 318 K, respectively.

It is noteworthy that the kinetic data for the initial stage of the oxidative polymerization of methylene blue are in satisfactory agreement with the first-order equation of the reaction rate (1).

(1) $$-\frac{{\mathrm{dC}}_{M}}{\mathrm{dt}}={\mathrm{kC}}_{M}$$

where: $C_{M}$—monomer concentration; t—time; k—reaction rate constant.

The concentration of the monomer reacted during the initial stage should be proportional to the change in the concentration of protons; therefore, the fulfillment of Equation (2) should be expected.

(2) $$C_{M}=\;X\left(C_{\max}-C\right)$$

where: C—current proton concentration; $C_{\max}$—maximum concentration of protons attained at the initial stage of the reaction; X—proportionality ratio.

After substitution of (2) into (1), separation of variables and subsequent integration from $C_{0}$ to C and from 0 to t leads to Equation (3).

(3) $$\ln\left(C_{\max}-C\right)=-\;\mathrm{kt}+\ln(C_{\max}-{\;C}_{0})$$

where: $C_{0}$—initial concentration of protons.

**Figure 2:**
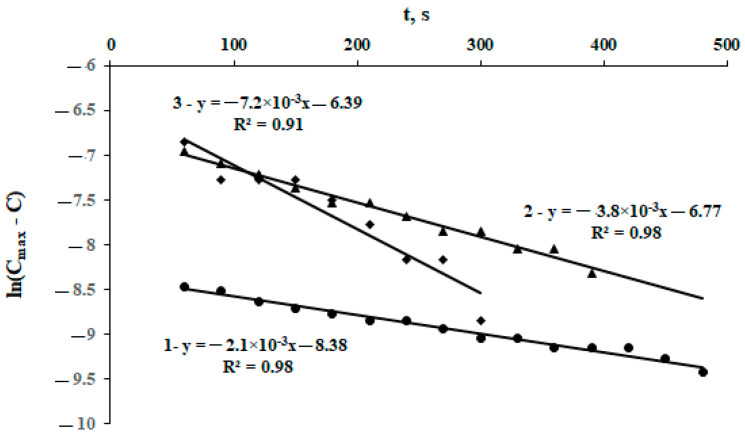
Linear anamorphoses of kinetic curves in coordinates “$\ln\left(C_{\max}-C\right)$ vs. t”, obtained for the polymerization of methylene blue (0.005 M) at the following temperatures: 1–298 K; 2–308 K; 3–318 K.

Kinetic Equation (3) satisfactorily agrees with the experimental data for the initial stage of methylene blue polymerization (Figure 2).

**Figure 3:**
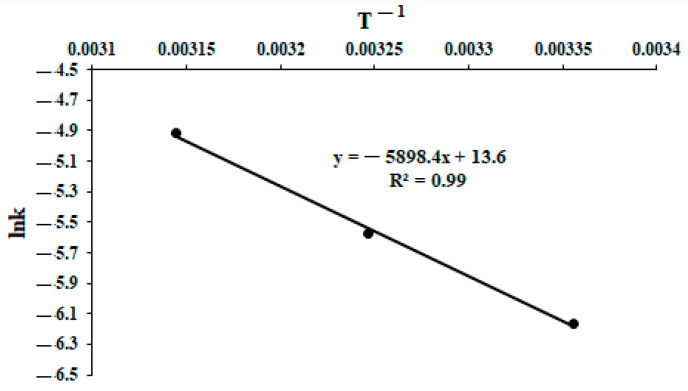
Temperature dependence of the rate constant of the oxidative polymerization of methylene blue in coordinates “lnk vs. T^−1^”.

The values of the rate constants determined from the tangents of the slope of straight lines in the coordinates “$\ln\left(C_{\max}-C\right)$ vs. t” are 2.1 × 10^−3^, 3.8 · 10^−3^, and 7.2 · 10^−3^ s^−1^ at temperatures of 298, 308, and 318 K, respectively. The temperature dependence of the rate constant of the oxidative polymerization of methylene blue is linear in the coordinates “lnk vs. T^−1^” (Figure 3). The activation energy for polymerization of methylene blue is 49 kJ · mol^−1^, and the preexponential factor is 7.9 · 10^5^ s^−1^.

**Scheme 1:**
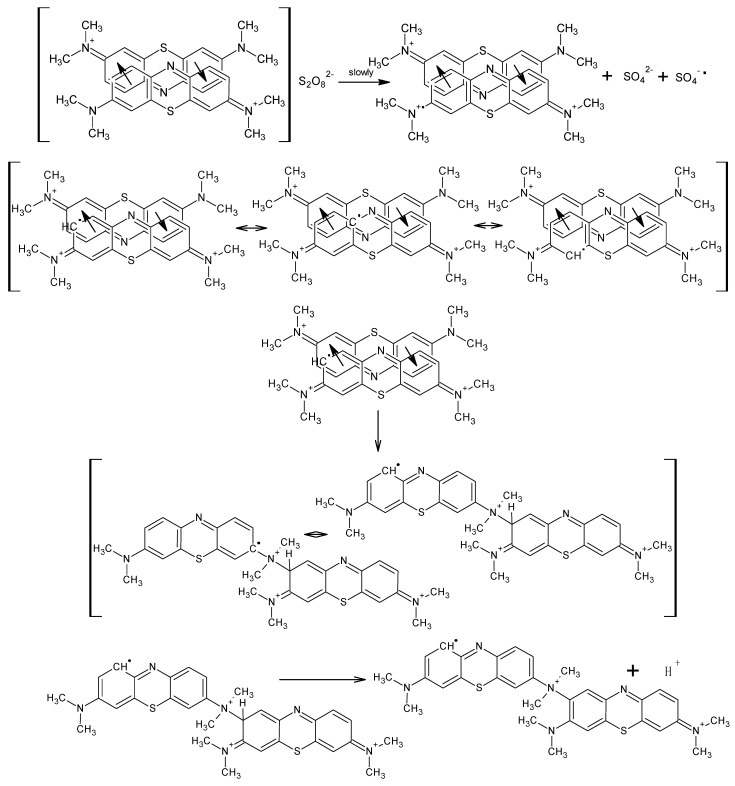
The mechanism of the initial stage of the oxidative polymerization of methylene blue.

It is known that methylene blue is capable of forming molecular associates in aqueous solutions, among which the dimeric form predominates [20]. The first order of the initial stage of polymerization of methylene blue is probably a consequence of the transformation of molecular dimers of methylene blue, stabilized by charge transfer, into cation radicals of dye dimers built by covalent bonds (Scheme 1). Apparently, the reaction begins with the one-electron oxidation of the methylene blue dimer at the dimethylamino group. The proposed mechanism of the initial stage of the oxidative polymerization of methylene blue with the participation of the most stable resonance structures is shown in Scheme 1. The values of the enthalpy of activation and entropy of activation, calculated in accordance with the theory of the activated complex, are 46.5 kJ · mol^−1^ and −140 J · mol^−1^ · K^−1^, respectively. The negative value of the activation entropy of the initial stage of polymerization of methylene blue indicates the compactness of the transition state in comparison with the reagents and is consistent with the slow oxidation of methylene blue within the ion pair formed by the molecular dimer of the dye (doubly charged cation) and the peroxodisulfate anion. The calculated activation parameters are consistent with the rapid approach of these ions so that the ion pair actually functions as a monomer. A similar case was described earlier for the oxidation of dopamine hydrochloride by the action of ammonium peroxodisulfate [21].

**Scheme 2:**
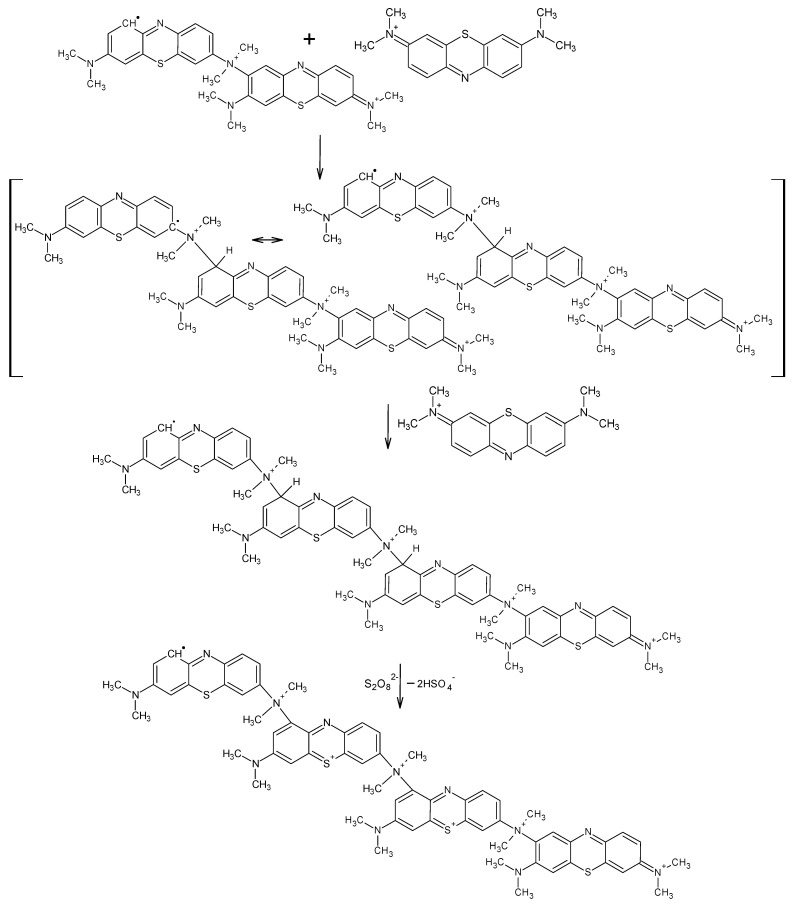
Mechanism of chain growth under conditions of methylene blue polymerization.

We assume that the subsequent formation of polymer chains is associated with the slow diffusion of methylene blue to the radical cations of methylene blue dimers, as shown in Scheme 2.

**Scheme 3:**
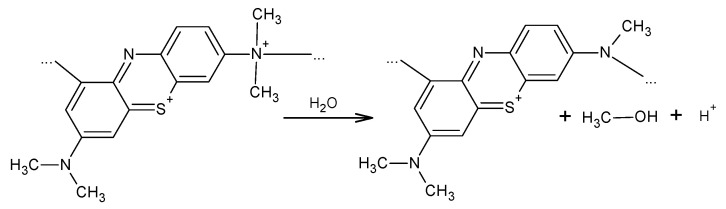
Possible structures of polymethylene blue chain links: nucleophilic demethylation.

Thus, considering the mechanism of methylene blue polymerization, one can expect the formation of dicationic units of the polymer chain, as well as monocationic fragments as a result of nucleophilic demethylation (Scheme 3).

**Scheme 4:**
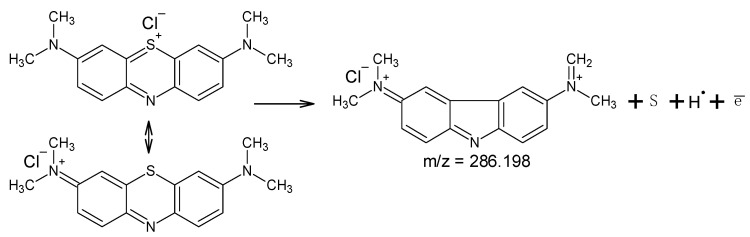
Destruction of methylene blue under conditions of laser desorption from a DHB matrix.

**Figure 4:**
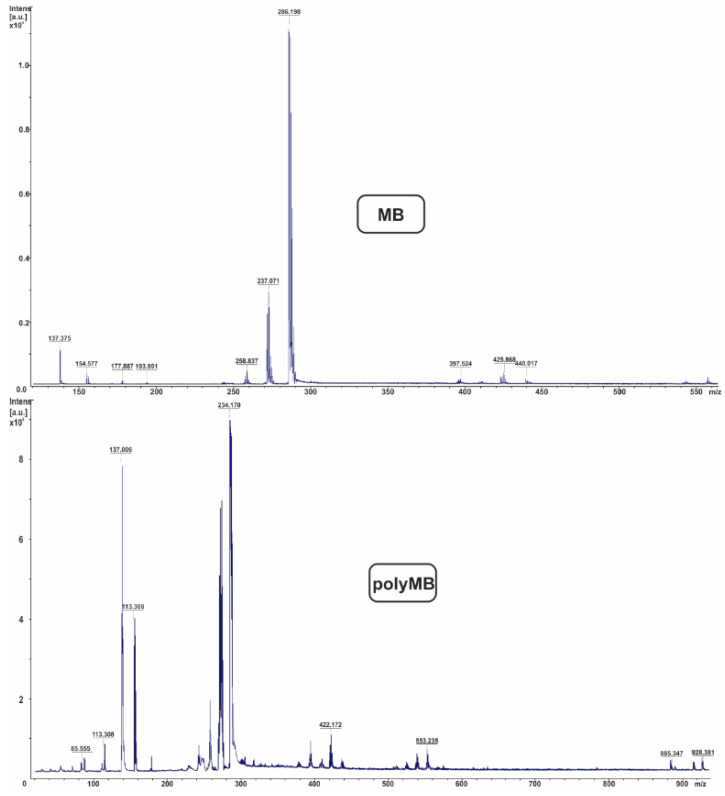
Spectra MALDI: MB—methylene blue; PolyMB—polymethylene blue.

The structure of the polymethylene blue chain is a subject of constant discussion in the literature and varies greatly in the works of various scientific teams [5,7,15,16,17]. Although the formation of quaternized nitrogen in the backbone seems to contradict the high electrical conductivity of polymethylene blue [15], the MALDI data indirectly indicate the presence of such units. In the MALDI of the starting methylene blue, under the conditions of detecting positively charged ions, signal 286.198 is observed (Figure 4), which, most likely, can be caused by the loss of sulfur and a hydrogen atom during laser desorption from the DHB matrix (Scheme 4).

**Scheme 5:**
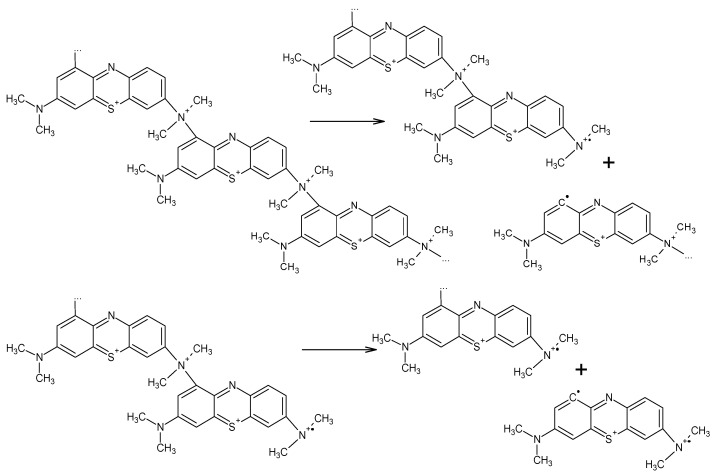
Destruction of the polymethylene blue chain under conditions of laser desorption from the DHB matrix.

For polymethylene blue under conditions of laser desorption from the DHB matrix, chain destruction can be expected in accordance with the mechanism presented in Scheme 5.

**Scheme 6:**
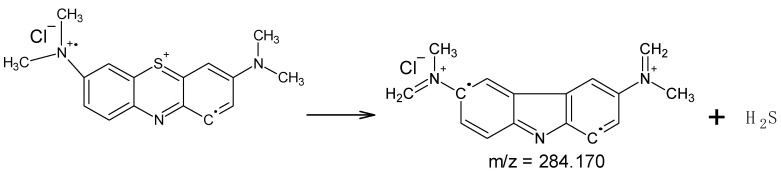
Extrusion of H_2_S by an intermediate formed during the destruction of the polymethylene blue chain because of laser desorption from the DHB matrix.

The pattern of the isotope distribution of the 284.17 signal (Figure 4) indicates the detection of ion pairs, which include the chloride anion. In this case, it can be expected that the reason for the most intense signal in MALDI is the loss of hydrogen sulfide by intermediates formed during chain destruction (Scheme 6).

The fraction of quaternized nitrogen in the main chain of polymethylene blue is probably small, and conjugation is realized in sections of the chain of considerable length. At the same time, even a small number of dicationic units can lead to a dominant signal in MALDI (Figure 4), because these fragments are probably responsible for electrostatic destabilization of the chain.

**Figure 5:**
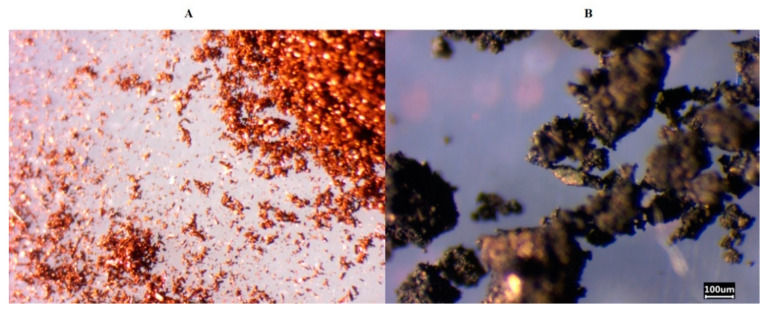
Photos of samples: (**A**)—methylene blue; (**B**)—polymethylene blue.

The above assumptions are in indirect agreement with reports on the high electrical conductivity of polymethylene blue [15], as well as with the pronounced metallic luster of this polymer (Figure 5).

**Figure 6:**
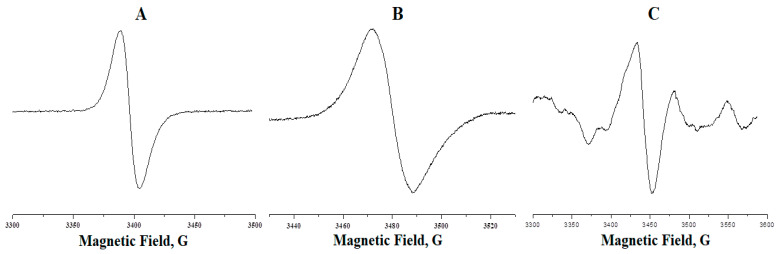
EPR spectra: (**A**)—methylene blue; (**B**)—polymethylene blue washed with five portions of distilled water, 100 mL each; (**C**)—washed with 25 portions of distilled water, 100 mL each.

The mechanism of the onset of electrical conductivity may possibly also include the inter-chain contribution associated with the formation of complexes with charge transfer. EPR spectroscopic data are consistent with this assumption. First, methylene blue, used as a monomer, has an EPR signal with a line width of ∆H = 15.9 G and a g-factor of ~2.0045 (Figure 6A). A polymethylene blue sample washed with five 100 mL portions of distilled water has an EPR signal with a width of ∆H = 16.6 G and a g-factor of ~2.0050 (Figure 6B). The close EPR spectra of methylene blue and polymethylene blue suggest that, in both cases, the signal is caused by the formation of a charge transfer complex [18,22,23,24,25]. Taking into account the known ability of methylene blue to adsorb on various electrically conductive polymers [26,27,28,29], and the effectiveness of polymethylene blue as an adsorbent [13], we can assume chemisorption of methylene blue on polymethylene blue is a result of the formation of charge transfer complexes with the polymer chain links. The broadening of the EPR signal in this case is caused by the possibility of the formation of a number of different, but structurally related, complexes with charge transfer. Thorough purification of polymethylene blue with 25 portions of distilled water, 100 mL each, leads to a decrease in intensity and an increase in the width of the EPR signal to ∆H = 19.5 G, which is consistent with the formation of complexes with charge transfer between the monomer and the links of the polymer chain (Figure 6C). In the latter case, the concentration of paramagnetic centers is about 5 · 10^16^ spin · g^−1^.

**Figure 7:**
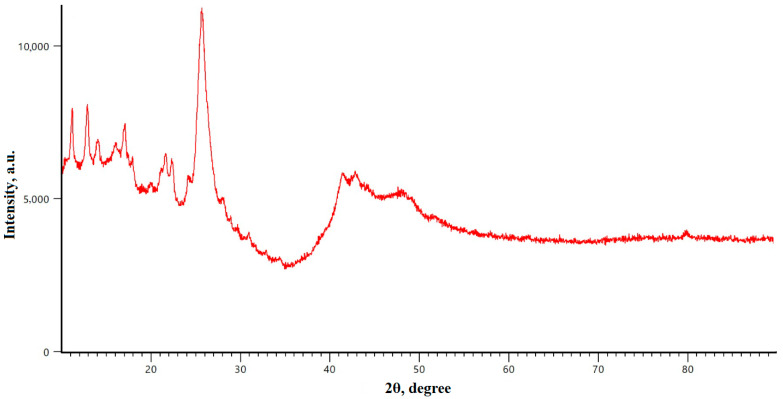
XRD of polymethylene blue synthesized by chemical oxidative polymerization of methylene blue.

According to XRD data, polymethylene blue resulting from the chemical oxidative polymerization of methylene blue contains a significant amount of amorphous phase (Figure 7).

**Figure 8:**
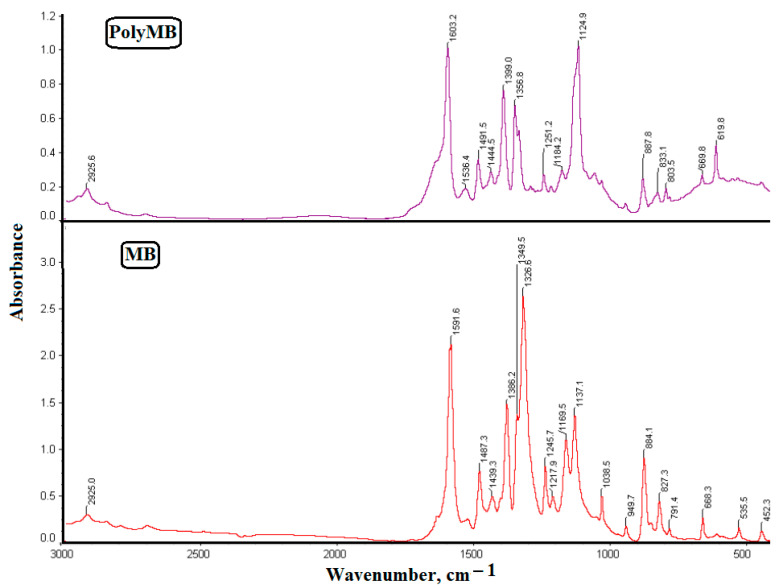
IR spectra: MB—methylene blue; PolyMB—polymethylene blue, recorded in potassium bromide tablets.

IR spectroscopic data indicate an increase in wavenumbers by 10–50 cm^−1^ for most of the stretching and bending vibration bands in the range 1000–1700 cm^−1^ after polymerization of methylene blue (Figure 8). This circumstance is consistent with the more “rigid” structure of polymethylene blue compared to the initial methylene blue, which exists predominantly in the form of a molecular dimer stabilized by charge transfer [22,23,24,25]. The appearance of a shoulder of skeletal vibrations at 1600.2 cm^−1^ in the region of high wavenumbers, a change in the position of the bands of out-of-plane bending vibrations of C-H bonds, and a decrease in their number are consistent with an increase in the resonance energy and the participation of C-H bonds in the oxidative polymerization of methylene blue.

**Figure 9:**
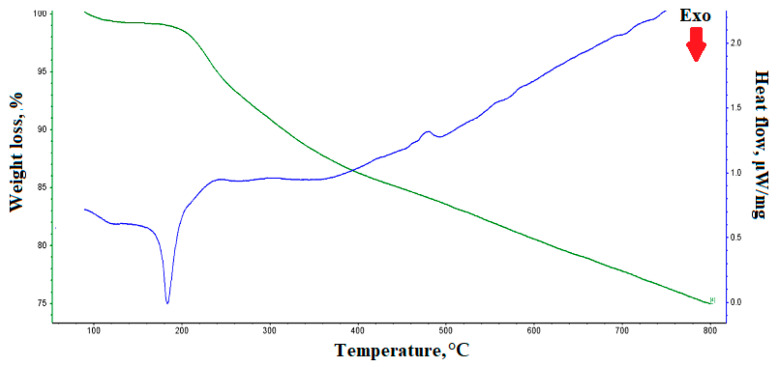
TGA and DSC of polymethylene blue.

If methylene blue begins to decompose slowly at temperatures above 373–383 K [30], then polymethylene blue, when heated to a temperature of about 440 K, undergoes only a loss of adsorbed water (Figure 9). It is not excluded that the exothermic peak in the temperature range 440–480 K, which corresponds to the constant mass of the sample, may be associated with rearrangements in the polymer chain, leading to the disappearance of energetically unfavorable dication units.

Although polymethylene blue is unstable when heated above 440 K, low-volatility compounds predominate among its decomposition products. Thus, when heated to 1000 K, the sample retains about 75 wt% of the initial mass.

## 4. Conclusions

It is shown that the rate of the initial stage of chemical oxidative polymerization of methylene blue obeys a first-order kinetic equation with an activation energy of 49 kJ · mol^−1^. The first kinetic order is presumably a consequence of the rapid convergence of the molecular dimer of methylene blue, stabilized by charge transfer, and the peroxodisulfate anion, followed by a slow one-electron transfer within the ion pair. The implementation of one-electron transfer within the ion pair is also indicated by the low value of the preexponential factor 7.9 · 105 s^−1^ and the negative value of the activation entropy −140 J · mol^−1^ · K^−1^. As a result, at the initiation stage, a radical cation of the covalent methylene blue dimer is formed, which is capable of subsequent addition of monomer molecules. Taking into account electronic and spatial factors, as well as kinetic data, a mechanism for the polymerization of methylene blue is proposed, which implies the formation of a chain linked through position 8 of the ring and quaternized nitrogen atoms, and the possibility of nucleophilic demethylation of the initially formed polymer is considered. The proposed structure of the polymethylene blue chain assumes the presence of extended conjugated sections of the chains and is consistent with its metallic luster and the data of MALDI, EPR, and IR spectroscopy methods. It was found that polymethylene blue can maintain stability up to 440 K; however, with a further increase in temperature up to 1000 K in an inert atmosphere, only 25 wt% of products are formed, subject to entrainment.

## Data Availability

The data presented in this study are available on request from the corresponding author.

## References

[1] Kaplan İ.H., Dağcı K., Alanyalıoğlu M. (2010). Nucleation and Growth Mechanism of Electropolymerization of Methylene Blue: The Effect of Preparation Potential on Poly(methylene blue) Structure. Electroanalysis.

[2] Liu B., Cang H., Cui L., Zhang H. (2017). Electrochemical Polymerization of Methylene Blue on Glassy Carbon Electrode. Int. J. Electrochem. Sci..

[3] Choi H.N., Lee W.Y. (2012). Amperometric Determination of Nitrite at Poly(Methylene Blue)-Modified Glassy Carbon Electrode. Bull. Korean Chem. Soc..

[4] Giribabu K., Suresh R., Manigandan R., Munusamy S., Kumar S.P., Muthamizh S., Narayanan V. (2013). Nanomolar determination of 4-nitrophenol based on a poly(methylene blue)-modified glassy carbon electrode. Analyst.

[5] Esokkiya A., Sudalaimani S., Kumar K.S., Sampathkumar P., Suresh C., Giribabu K. (2021). Poly(methylene blue)-Based Electrochemical Platform for Label-Free Sensing of Acrylamide. ACS Omega.

[6] Marinho M.I.C., Cabral M.F., Mazo L.H. (2012). Is the poly(methylene blue)-modified glassy carbon electrode an adequate electrode for the simple detection of thiols and amino acid-based molecules?. J. Electroanal. Chem..

[7] Manasa G., Mascarenhas R.J., Satpati A.K., D’Souza O.J., Dhason A. (2017). Facile preparation of poly(methylene blue) modified carbon paste electrode for the detection and quantification of catechin. Mater. Sci. Eng. C.

[8] Tan L., Xie Q., Yao S. (2004). Electrochemical and Spectroelectrochemical Studies on Pyridoxine Hydrochloride Using a Poly(methylene blue) Modified Electrode. Electroanalysis.

[9] Xiaoqin L., Zhang P., Li M., Guo Z., Zheng X. (2016). Synthesis of Polymethylene Blue Nanoparticles and Their Application to Label-Free DNA Detection. Anal. Lett..

[10] Brett C.M.A., Inzelt G., Kertesz V. (1999). Poly(methylene blue) modifed electrode sensor for haemoglobin. Anal. Chim. Acta.

[11] Liu G., Ma W., Luo Y., Sun D., Shao S. (2014). Simultaneous Determination of Uric Acid and Xanthine Using a Poly(Methylene Blue) and Electrochemically Reduced Graphene Oxide Composite Film Modified Electrode. J. Anal. Methods Chem..

[12] Braun W.A., Horn B.C., Hoehne L., Stülp S.R., Marcelo B.D., Hilgemann M. (2017). Poly(methylene blue)-modified electrode for indirect electrochemical sensing of OH radicals and radical scavengers. An. Acad. Bras. Cien..

[13] Wang N., Xiao S.J., Su C.W. (2016). Preparation of molecularly imprinted polymer for methylene blue and study on its molecular recognition mechanism. Colloid Polym. Sci..

[14] Samyn L.M., Babu R.S., Devendiran M., de Barros A.L.F. (2021). One-step electropolymerization of methylene blue films on highly flexible carbon fiber electrode as supercapacitors. Micro Nano Syst. Lett..

[15] Hichem H., Djamila A., Hania A. (2013). Optical, electrical and photoelectrochemical characterization of electropolymerized poly methylene blue on fluorine doped tin oxide conducting glass. Electrochim. Acta.

[16] Ajami N., Ehsani A., Babaei F., Safari R. (2016). Electrochemical properties, optical modeling and electrocatalytic activity of pulse-electropolymerized ternary nanocomposite of poly(methylene blue) in aqueous solution. J. Mol. Liq..

[17] Liu J., Mu S. (1999). The electrochemical polymerization of methylene blue and properties of polymethylene blue. Synth. Met..

[18] Kamiya N., Okawara M. (1969). Photopolymerization Using Methylene Blue-Reducing Agent System and Its Application to Photoelectrochemical Cell. J. Soc. Chem. Ind. Jpn..

[19] Fouassier J.-P., Morlet-Savary F., Lalevée J., Allonas X., Ley C. (2010). Dyes as Photoinitiators or Photosensitizers of Polymerization Reactions. Materials.

[20] Cenens J., Schoonheydt R.A. (1988). Visible Spectroscopy of Methylene Blue on Hectorite, Laponite B, and Barasym in Aqueous Suspension. Clays Clay Miner..

[21] Mezhuev Y.O., Varankin A.V., Luss A.L., Dyatlov V.A., Tsatsakis A.M., Stratidakis A.K., Korshak Y.V. (2020). Abnormally slow reaction of oppositely charged ions: The kinetics of dopamine hydrochloride oxidation by ammonium peroxydisulfate. Int. J. Chem. Kinet..

[22] Erickson P.R., Walpen N., Guerard J.J., Eustis S.N., Arey J.S., McNeill K. (2015). Controlling Factors in the Rates of Oxidation of Anilines and Phenols by Triplet Methylene Blue in Aqueous Solution. J. Phys. Chem. A.

[23] Kayser R.H., Young R.H. (1976). The Photoreduction of Methylene Blue by Amines-I. A Flash Photolysis Study of The Reaction Between Triplet Methylene Blue and Amines. Photochem. Photobiol..

[24] Murthy A.S.N., Bhardwaj A.P. (1980). Electronic absorption spectroscopic studies on the red form of methylene blue ion. Proc. Indian Acad. Sci. Chem. Sci..

[25] Junqueira H.C., Severino D., Dias L.G., Gugliotti M.S., Baptista M.S. (2002). Modulation of methylene blue photochemical properties based on adsorption at aqueous micelle interfaces. Phys. Chem. Chem. Phys..

[26] Mohammad N., Atassi Y. (2020). Adsorption of methylene blue onto electrospun nanofibrous membranes of polylactic acid and polyacrylonitrile coated with chloride doped polyaniline. Sci. Rep..

[27] Ayad M.M., El-Nasr A.A. (2010). Adsorption of Cationic Dye (Methylene Blue) from Water Using Polyaniline Nanotubes Base. J. Phys. Chem. C.

[28] Maruthapandi M., Kumar V.B., Luong J.H.T., Gedanken A. (2018). Kinetics, Isotherm, and Thermodynamic Studies of Methylene Blue Adsorption on Polyaniline and Polypyrrole Macro-Nanoparticles Synthesized by C-Dot-Initiated Polymerization. ACS Omega.

[29] Ayad M.M., Amer W.A., Zaghlol S., Minisy I.M., Bober P., Stejskal J. (2018). Polypyrrole-coated cotton textile as adsorbent of methylene blue dye. Chem. Pap..

[30] (1978). Kirk-Othmer Encyclopedia of Chemical Technology.

